# Cervical lymphoepithelioma-like carcinoma with deficient mismatch repair and loss of SMARCA4/BRG1: a case report and five related cases

**DOI:** 10.1186/s13000-023-01429-2

**Published:** 2024-01-04

**Authors:** Yu Miyama, Tomomi Kato, Masayasu Sato, Akira Yabuno, Kosei Hasegawa, Masanori Yasuda

**Affiliations:** 1 Department of Pathology, Saitama Medical University International Medical Center, 1397-1, Yamane, Hidaka, Saitama 350-1298 Japan; 2 Department of Gynecologic Oncology, Saitama Medical University International Medical Center, 1397-1, Yamane, Hidaka, Saitama 350-1298 Japan

**Keywords:** Lymphoepithelioma-like carcinoma, Medullary carcinoma, Mismatch repair, Programmed cell death ligand-1, Human papillomavirus-associated, Squamous cell carcinoma, Switch/sucrose non-fermentable family complex

## Abstract

**Background:**

We encountered a cervical lymphoepithelial carcinoma (LEC) possessing a predominantly solid architecture with deficient mismatch repair (dMMR) and loss of expression of the SWI/SNF (SWItch/Sucrose Non-Fermentable) chromatin remodeling complex subunit. This is the first case report of LEC with dMMR and loss of SWI/SNF complex subunit.

**Case presentation:**

A 34-year-old woman presented at our hospital with menstrual irregularities and abnormal vaginal bleeding. Magnetic resonance imaging revealed an exophytic mass in the posterior uterine cervix. Biopsy specimens confirmed squamous cell carcinoma with a 2018 International Federation of Gynecology and Obstetrics (FIGO) uterine cervical cancer stage of IB2. In a subsequent conization specimen, the tumor appeared exophytic. Microscopically, the tumor cells formed a predominant solid architecture. Abundant lymphocytic infiltration was observed. The pathological diagnosis indicated human papillomavirus (HPV)-associated squamous cell carcinoma with LEC pattern and pT1b2. Immunohistochemically, high programmed death-ligand 1 (PD-L1) expression, dMMR, and loss of the switch/sucrose non-fermentable family-related, matrix-associated, actin-dependent regulator of chromatin subfamily member 4 (SMARCA4)/BRG1, an SWI/SNF complex subunit, were observed. The patient underwent a radical hysterectomy and is alive without disease one year and five months later. Our analysis of five additional LEC cases revealed a consistent association with high-risk HPV and elevated PD-L1 expression. In addition to the present case, another patient exhibited dMMR. The SWI/SNF complex was retained except in the present case. The prognosis was favorable in all cases.

**Conclusions:**

This unique case of LEC with dMMR suggests a distinct clinical entity with potential immunotherapy implications. Analysis of the other five LEC cases revealed that LEC was immune hot, and immune checkpoint inhibitors may be effective. The two dMMR cases showed loss of MLH1 and PMS2 expressions, and prominently high tumor PD-L1 expression. In those cases, dMMR might have contributed to the morphological characteristics of LEC.

**Supplementary Information:**

The online version contains supplementary material available at 10.1186/s13000-023-01429-2.

## Background

Cervical lymphoepithelial carcinoma (LEC) is a rare histological pattern of uterine cervix squamous cell carcinoma associated with the high-risk human papillomavirus (HPV) [1–4]. LEC is characterized by marked lymphocytic infiltration, suggesting the potential effectiveness of immune checkpoint inhibitors [5–7]. Nasopharyngeal and gastric LECs are linked to the Epstein–Barr virus (EBV) [8–10], while cervical LECs are not [1–4]. Therefore, we hypothesized the presence of mechanisms that enhance tumor immunity. Here, we report the first case of cervical LEC with high PD-L1 expression and deficient mismatch repair (dMMR). In addition, the tumor exhibited a solid architecture. This solid architecture with lymphocytic infiltration is reminiscent of colorectal medullary carcinoma and solid-type poorly differentiated gastric adenocarcinoma, both of which have dMMR and a deficient SWF/SNF complex [11–13]. In the present case, SMARCA4/BRG1, a subunit of the SWF/SNF complex, demonstrated a loss of expression. Additionally, we present the histological and immunophenotypic results of five other cases of LEC.

## Case presentation

### Clinical findings

**Fig. 1:**
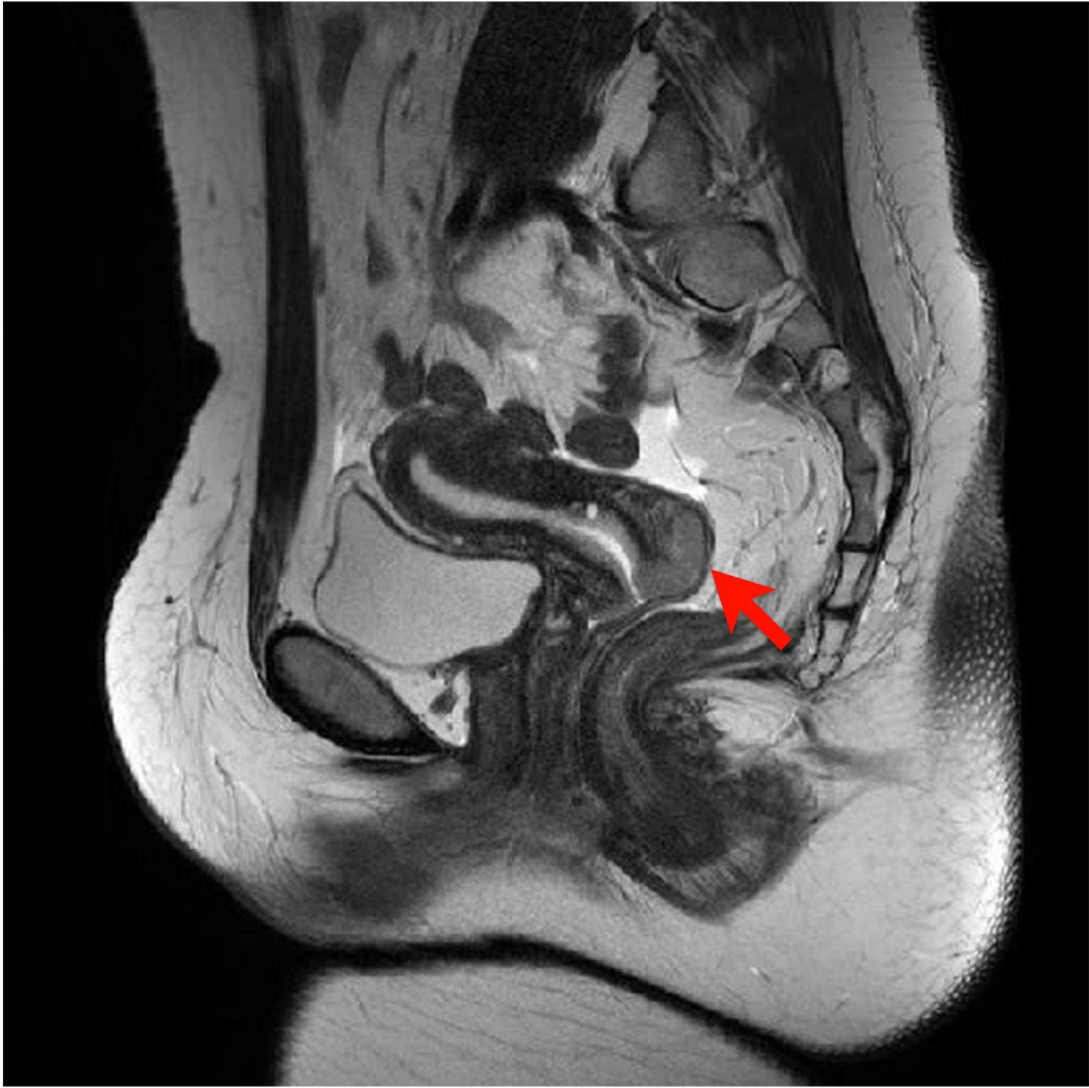
Magnetic resonance imaging. An exophytic mass is more prominent in the T2-weighted image in the posterior uterine cervix (arrow)

A 34-year-old woman presented to our hospital with menstrual disorders and abnormal vaginal bleeding. She was obese, had a history of type 2 diabetes mellitus and hyperlipidemia, and reported having had sexual intercourse but with no history of pregnancy. Magnetic resonance imaging revealed an exophytic mass in the posterior cervix (Fig. 1). Biopsy specimens confirmed squamous cell carcinoma, staging it as IB2 per the International Federation of Gynecology and Obstetrics (FIGO) 2018 uterine cervical cancer staging system. Subsequent conization was performed.

### Pathological findings of conization specimen

**Fig. 2:**
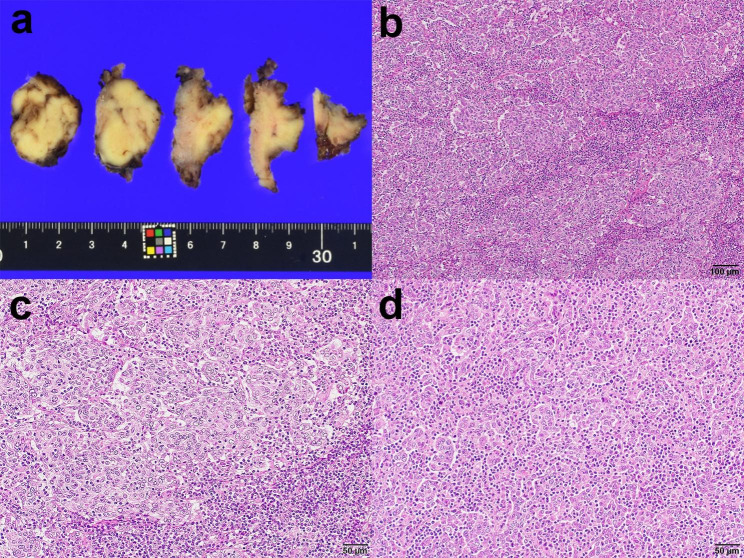
Pathological findings of the conization specimen in the present lymphoepithelioma-like carcinoma case. (**a**) The tumor is a whitish and exophytic lesion with a 3.2-cm maximum diameter. (**b**) The tumor predominantly exhibits a solid architecture. (**c**) High-power view of the solid architecture shows marked lymphocytic infiltration. (**d**) Focally, a reticular architecture with lymphocytic infiltration, described as a lace-like pattern, can also be noted

Macroscopically, the tumor appeared as a whitish exophytic lesion with a maximum diameter of 3.2 cm. Microscopically, tumor cells with irregular and hyperchromatic nuclei formed a solid, reticular architecture with stromal invasion (Fig. 2). High-grade squamous intraepithelial lesions (HSIL/CIN3) were also observed. In the stromal invasion area, abundant lymphocytic infiltration was noted. The pathological diagnosis indicated squamous cell carcinoma (lymphoepithelioma-like carcinoma), HPV-associated, and pT1b2. Lymphatic permeation was also observed.

**Fig. 3:**
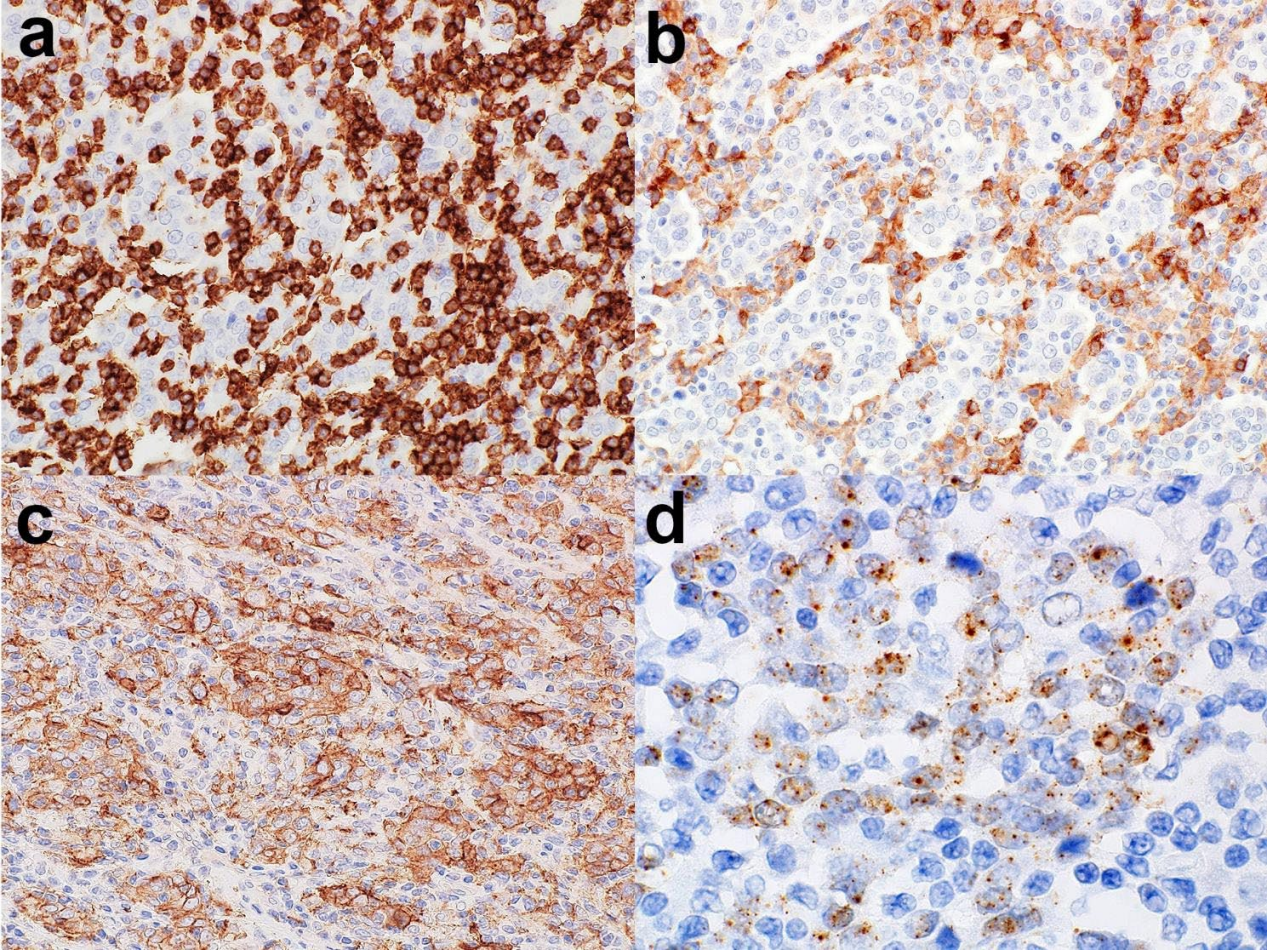
Tumor immune microenvironment and high-risk human papillomavirus (HPV) infection status. (**a**) CD8-positive lymphocytes are densely arranged in the tumor and stroma. (**b**) CD4-positive lymphocytes can be observed primarily in the stroma. (**c**) Programmed cell death ligand-1 expression is detectable not only in tumor cells but also in inflammatory cells. The total positive score was 80. (**d**) High-risk HPV RNA in situ hybridization. Dot-like signals can be observed in tumor cells, suggesting viral gene integration into the tumor genome

Immunohistochemically, the tumor cells were positive for p63, p40, and p16. The infiltrative lymphocytes were predominantly CD8-positive, with scattered CD4-positive or CD20-positive lymphocytes (Fig. 3a and b). The total positive score (TPS) of PD-L1 (programmed cell death-ligand 1) was 80 (Fig. 3c), according to a scoring algorithm [14].

**Fig. 4:**
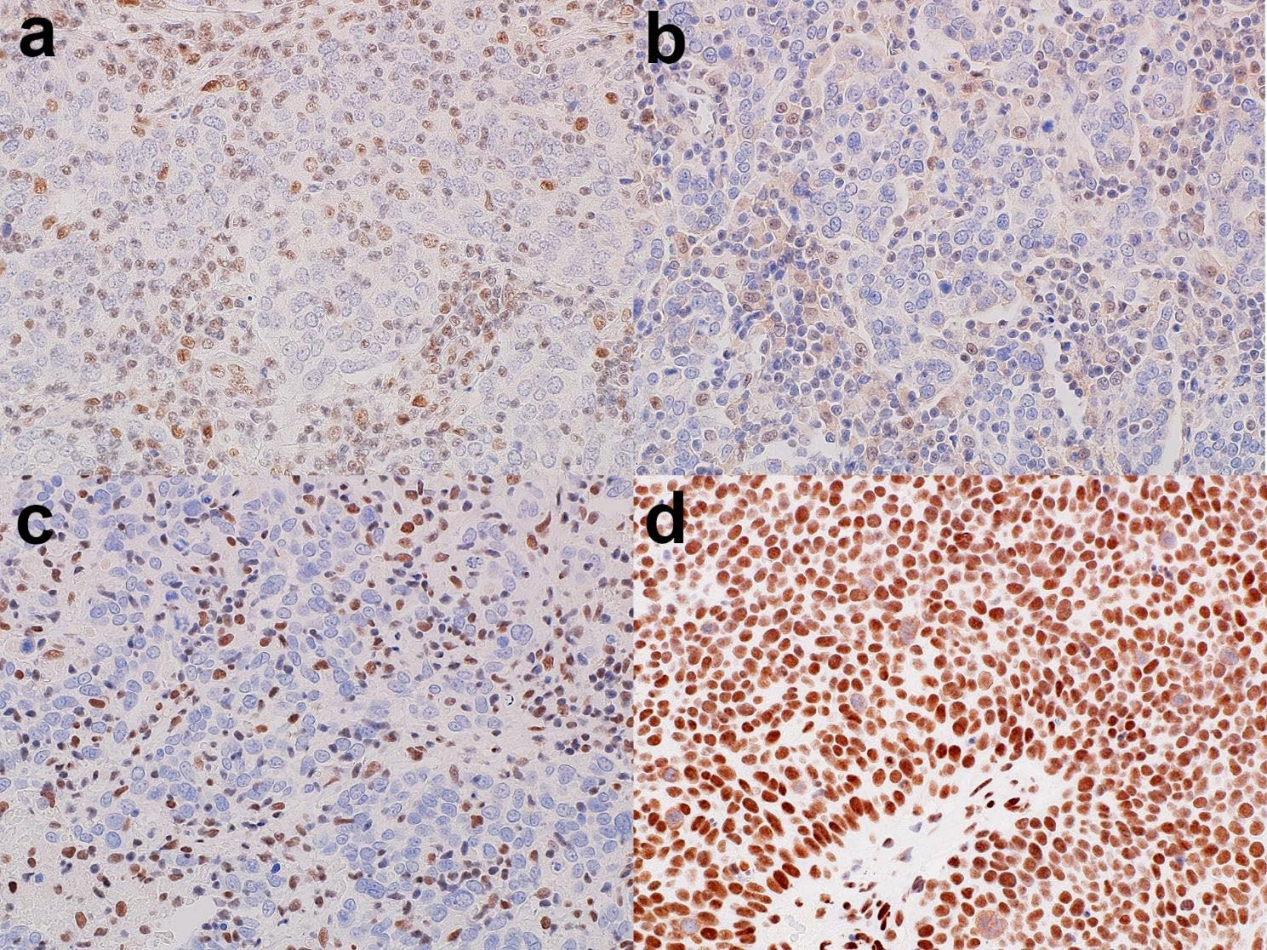
Mismatch repair and SWI/SNF complex status. MLH1 (**a**) and PMS2 (**b**) are negative in tumor cells, suggesting deficient mismatch repair. BRG1 is negative in invasive carcinoma (**c**), whereas BRG1 expression is retained in high-grade intraepithelial squamous lesions (**d**)

In addition, the tumor cells were negative for MLH1 and PMS2, suggesting dMMR, as shown in Fig. 4a and b. Moreover, the tumor cells were negative for SMARCA4/BRG1 in invasive carcinoma but not in HSIL/CIN3 (Fig. 4c and d).

High-risk HPV RNA was detected using in situ hybridization (Fig. 3d). EBV-encoded small RNA (EBER) was not detected.

### Patient’s outcome

The patient underwent a radical hysterectomy. Only HSIL/CIN3 remained in the residual uterine cervix. HSIL/CIN3 showed proficient mismatch repair (MMR) (Supplementary Fig. 1). Pelvic sentinel lymph nodes were negative for malignancy, and lymphadenectomy was omitted. She is alive without disease one year and five months after the hysterectomy.

### The present case and five other LEC cases

We analyzed five other LEC cases. Table 1 summarizes the pathological findings and patient outcomes, including those in the present case. Three tumors in the cases appeared exophytic. Microscopically, solid architecture was predominantly observed in three cases. All tumors exhibited marked CD8-positive lymphocytic infiltration. In addition to the present case, another case had dMMR with the loss of MLH1 and PMS2 expressions (case 2, Supplementary Fig. 2). Although all cases showed high PD-L1 TPS, it was particularly high among the dMMR cases. The SWI/SNF complex subunits were retained, except in the present case. High-risk HPV was detected in all tumors. The prognosis was generally favorable.

**Table 1:** Pathological findings in six LEC cases

Case	Age (years)	Specimen	Gross appearance	Architecture	MMR status *	SWI/SNF complex subunit **	CD4	CD8	CD20	PD-L1 TPS	High-risk HPV ISH***	EBER ISH	FIGO 2018 Stage	Initial treatment	Outcome
Present case 1	34	Conization	Exophytic	Solid > Lace-like	Deficient (MLH1, PMS2)	Lost (BRG1)	Mild	Marked	Mild	80	+ (RNA)	-	IB2	Conization and Hysterectomy	NED, 1 year 5 months
2	39	Biopsy	Endophytic	Lace-like > Solid	Deficient (MLH1, PMS2)	Retained	Moderate	Marked	Mild	60	+ (RNA)	-	IIIC1	CCRT	NED, 4 years
3	60	Biopsy	Endophytic	Solid	Proficient	Retained	Mild	Marked	Mild	10	+ (DNA)	-	IIB	CCRT	NED, 6 years
4	80	Excisional biopsy	Exophytic	Lace-like > solid	Proficient	Retained	Mild	Marked	Mild	10	+ (DNA)	-	IB1	None	Vaginal recurrence, 6 years
5	68	Biopsy	Endophytic	Lace-like	Proficient	Retained	Moderate	Marked	Mild	15	+ (DNA)	-	IIB	CCRT	NED, 4 years 5 months
6	68	Biopsy	Exophytic	Solid	Proficient	Retained	Mild	Marked	Mild	10	+ (DNA)	-	IB1	None	Unknown

CCRT, concomitant chemoradiotherapy; EBER, Epstein-Barr virus-encoded small RNA; FIGO, International Federation of Gynecology and Obstetrics; PD-L1, programmed cell death-ligand 1; TPS, total positive score; SMARCA4, switch/sucrose non-fermentable family related, matrix associated, actin dependent regulator of chromatin, subfamily, member 4; SWI/SNF, switch/sucrose non-fermentable

* Immunohistochemical results for MLH1, PMS2, MSH2, and MSH6

** Immunohistochemical results of BRG1/SMARCA4, INI1/SMARCB1 and ARID1A

*** TPS was assessed based on The VENTANA PD-L1 (SP263) Scoring Algorithm (VENTANA PD-L1 (SP263) Assay Staining of Non-Small Cell Lung Cancer Interpretation Guide

**** DNA or RNA

## Discussion and conclusions

We encountered a case of cervical LEC with predominantly solid architecture. In addition to high-risk HPV association and high PD-L1 expression, dMMR and loss of SMARCA4/BRG1 were detected. We reported the status of five other LEC cases, as shown in Table 1.

High-risk HPV was detected in all the cases using high-risk HPV DNA or RNA in situ hybridization (ISH). Squamous intraepithelial lesions were also observed in the present case. In contrast, EBER-ISH results were negative in all cases. An association between LEC and EBV has been suggested [15, 16]. However, recently, an association between LEC and high-risk HPV, not EBV, has been suggested [1–4]. Cervical LEC is now classified as one of the histological patterns in HPV-associated squamous cell carcinoma [17].

Microscopically, the architecture of cervical LECs is described using variable pathological terminologies, such as poor, undifferentiated, sheet-like, and syncytial [3, 4, 15, 18, 19], primarily with marked lymphocytic infiltration. In the morphological analysis, both solid and lace-like patterns were observed (Table 1). In the present case, a solid pattern was predominant.

Tumors with microsatellite instability often show marked intratumoral and peri-tumoral lymphocytic infiltration [20, 21]. Therefore, we investigated the MMR status in LEC cases. In this study, two cases (33.3%), including the present case, showed dMMR. This frequency was higher than previously reported, 11.3% (21/186) of cervical cancers [22]. Meanwhile, Pinto et al. reported seven cases of cervical LEC, and all tumors showed proficient MMR [3]. Our two dMMR cases lost MLH1 and PMS2 expressions. Although we did not perform MLH1 promotor methylation analysis or MMR somatic/germline mutational analysis considering ethics, dMMR may be attributed to epigenetic MLH1 inactivation. In addition, in the present case, residual HSIL/CIN3, observed in residual uterine cervix of radical hysterectomy specimen, retained MMR. Similarly, coexistent HSIL/CIN3 retained MMR in case 2. We speculate that some HSIL/CIN3 cases obtain dMMR caused by epigenetic MLH1 inactivation, and enhance lymphocytic infiltration during tumor progression and are recognized as LEC. However, further accumulation of LEC cases and investigation of MMR status are needed, and the potential correlation between HPV infection and MMR in tumor progression could be explored.

In the present study, we also found an association between high PD-L1 expression and cervical LEC. Similarly, Pinto et al. reported that most LEC cases show high PD-L1 expression [3]. The prognostic value of PD-L1 expression in patients with invasive cervical carcinoma has been reported [23]. Recently, the US Food and Drug Administration (FDA) approved pembrolizumab for persistent, recurrent, or metastatic cervical cancer with a combined positive score ≥ 1, in addition to chemotherapy with or without bevacizumab [24]. In addition, the FDA also approved cemiplimab for the treatment of patients with recurrent or metastatic cervical cancer regardless of PD-L1 expression status [25]. Furthermore, as in the present case, dMMR is a predictive biomarker for the response to immune checkpoint inhibitors [22]. Currently, the two dMMR cases exhibited prominently high tumor PD-L1 expression. Although cervical LECs tend to have a favorable prognosis, patients with persistent cervical LECs are candidates for pembrolizumab treatment.

The SWI/SNF complex interacts with histones and transcription factors to modulate chromatin structure and gene expression [26]. The association of solid-type histology with dMMR and the SWI/SNF complex is well-known in colorectal and gastric cancer [11–13]. In the present case, a solid architecture was predominant, and we considered the possibility of deficiency in the SWI/SNF complex. The present case exhibited a loss of SMARCA4/BRG1 expression, a subunit of the SWI/SNF complex. Meanwhile, SMARCA4/BRG1 expression was retained in HSIL/CIN3. The deficiency of the SWI/SNF complex occurs at different stages of tumor development and is restricted to unique histologic types of gynecologic cancer [27]. In the uterine cervix, a deficient SWI/SNF complex was detected in 21.8% of 430 whole-exome-sequenced cervical cancers [28]. Considering its frequency, a deficient SWI/SNF complex may not be characteristic of cervical LEC. Nevertheless, we need to explore histological architecture, MMR status andalteration of *SMARCA4/BRG1* in cervical cancer.

In conclusion, we encountered a case of cervical LEC with a predominantly solid architecture, marked lymphocytic infiltration, high PD-L1 expression, dMMR, and loss of SMARCA4/BRG1 expression. Including five additional LEC cases, all cases were associated with high-risk HPV and high tumor PD-L1 expression; another case had dMMR. LECs exhibited a hot immune status, and immune checkpoint inhibitors may be effective; the prognosis was favorable. Unlike gastric or nasopharyngeal LEC, cervical LEC does not always form a predominant lace-like pattern or is not associated with EBV. Given the morphology or carcinogenesis, the histological pattern of LEC would be a misnomer. Nevertheless, recognition of this histological pattern is valuable for prognosis and therapeutic strategies. Although the level of lymphocytic infiltration remains to be classified as LEC is not determined, considering its possibility and checking PD-L1 and MMR status are important.

### Electronic supplementary material

Below is the link to the electronic supplementary material.

**Supplementary Material 1: : Fig 1.** Mismatch repair status of the radical hysterectomy specimen in the present case. Only high-grade squamous intraepithelial lesion was remaining (a). MLH1 (b) and PMS2 (c) expressions were retained, suggesting proficient mismatch repair

**Supplementary Material 2: : Fig 2.** Mismatch repair status of case 2. The tumor cells show solid architecture with lymphocytic infiltrations (a). MLH1 (b) and PMS2 (c) are negative in invasive carcinoma, suggesting deficient mismatch repair. However, in the high-grade squamous intraepithelial lesion (a, arrows), the MLH1 (e) and PMS2 (b) expressions are retained

## Data Availability

For ethical reasons, the data will only be made available upon reasonable request. Please contact us via email at miyamay@saitama-med.ac.jp.

## List of abbreviations

CIN3: cervical intraepithelial neoplasm 3
dMMR: deficient mismatch repair
EBV: Epstein-Barr virus
EBER: Epstein-Barr virus-encoded small RNA
FDA: the Food and Drug Administration
FIGO: International Federation of Gynecology and Obstetrics
HSIL: high-grade squamous intraepithelial lesion
LEC: lymphoepithelial carcinoma
MMR: mismatch repair
PD-L1: programmed cell death-ligand 1
TPS: total positive score
SMARCA4: switch/sucrose non-fermentable family related, matrix associated, actin-dependent regulator of chromatin, subfamily, member 4
SWI/SNF: SWItch/Sucrose Non-Fermentable family
