# Supplementary figures and images for: Cervical lymphoepithelioma-like carcinoma with deficient mismatch repair and loss of SMARCA4/BRG1: a case report and five related cases
Source: Diagn Pathol. 2024 Jan 4;19:6. doi: 10.1186/s13000-023-01429-2 (PMC10765828; doi:10.1186/s13000-023-01429-2)

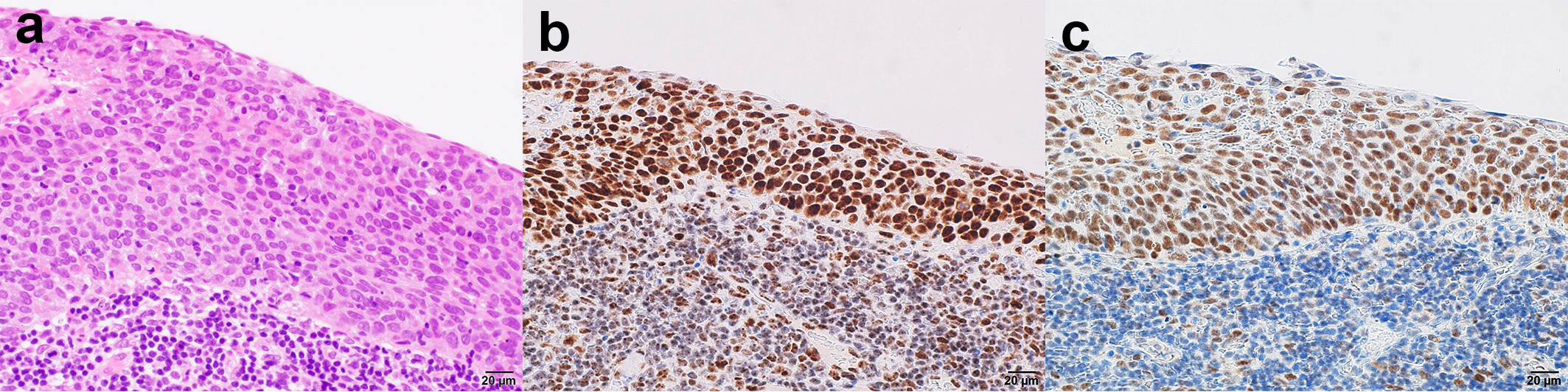

Supplement: Supplementary file 1 — Supplementary Material 1: : Fig 1. Mismatch repair status of the radical hysterectomy specimen in the present case. Only high-grade squamous intraepithelial lesion was remaining (a). MLH1 (b) and PMS2 (c) expressions were retained, suggesting proficient mismatch repair [file 13000_2023_1429_MOESM1_ESM.jpg]

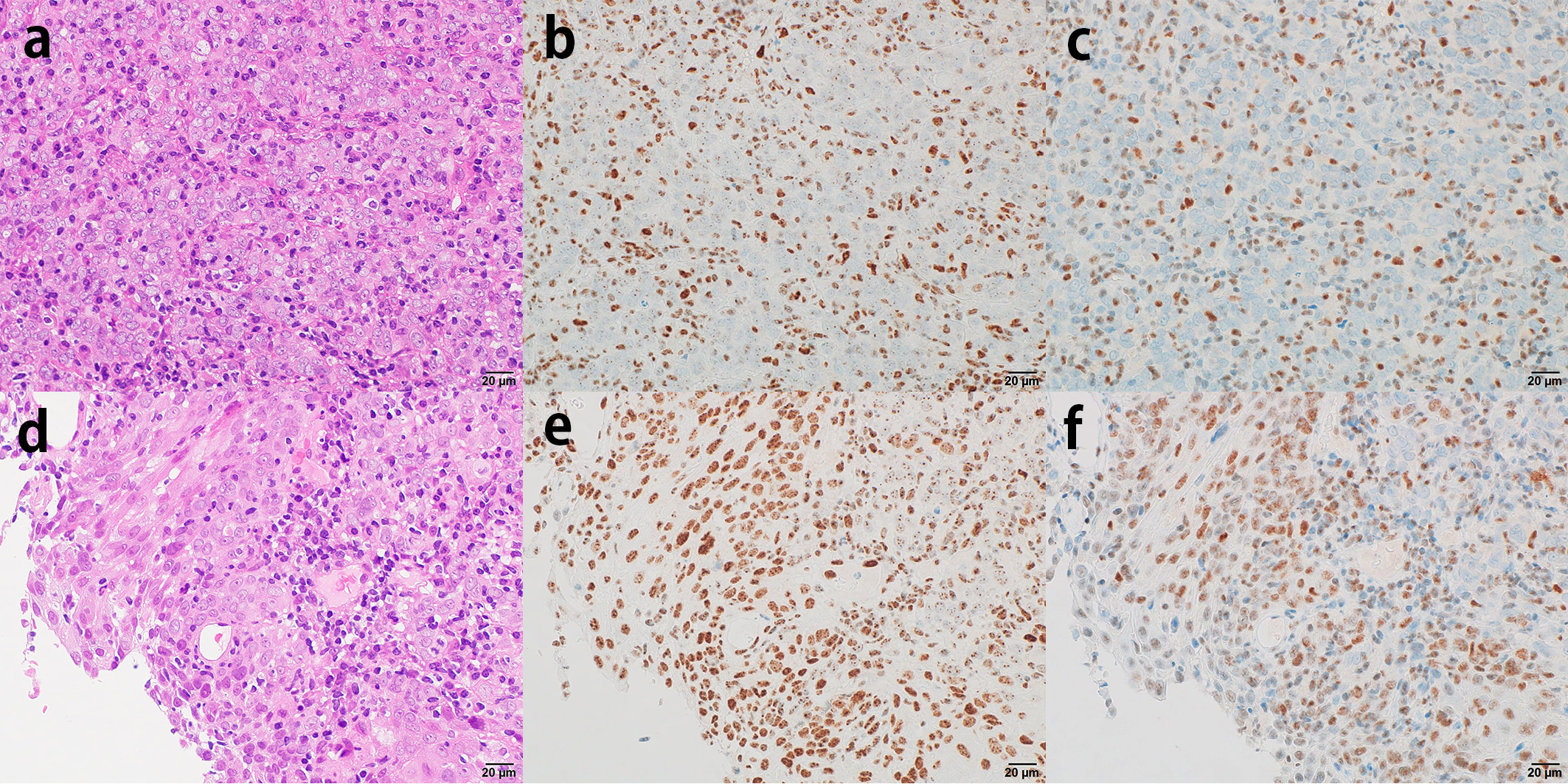

Supplement: Supplementary file 2 — Supplementary Material 2: : Fig 2. Mismatch repair status of case 2. The tumor cells show solid architecture with lymphocytic infiltrations (a). MLH1 (b) and PMS2 (c) are negative in invasive carcinoma, suggesting deficient mismatch repair. However, in the high-grade squamous intraepithelial lesion (a, arrows), the MLH1 (e) and PMS2 (b) expressions are retained [file 13000_2023_1429_MOESM2_ESM.jpg]
